# Associations of sedentary behavior and physical activity with physical measurements and dyslipidemia in school-age children: a cross-sectional study

**DOI:** 10.1186/s12889-016-3826-y

**Published:** 2016-11-24

**Authors:** Wei Zheng, Yun Chen, Ai Zhao, Yong Xue, Yingdong Zheng, Zhishen Mu, Peiyu Wang, Yumei Zhang

**Affiliations:** 1 Department of Social Medicine and Health Education, School of Public Health, Peking University Health Science Center, Beijing, China; 2 Dairy Research Institute, Inner Mongolia Mengniu Dairy (Group) Co. Ltd, Inner mongolia, China; 3 CAS key Laboratory of Pathogenic Microbiology and Immunology, Institute of Microbiology, Chinese Academy of Science, Beijing, China; 4 Department of Epidemiology and Biostatistics, School of Public Health, Peking University Health Science Center, Beijing, China; 5 Department of Nutrition and Food Hygiene, School of Public Health, Peking University Health Science Center, Beijing, China; 6 Beijing Key Laboratory of Toxicological Research and Risk Assessment for Food Safety, Beijing, China

**Keywords:** Dyslipidemia, Anthropometric measurements, Physical activity, Sedentary behavior

## Abstract

**Background:**

Physical activity and sedentary behavior are common factors influencing cardiovascular health. However, how school and leisure-time activity/sedentary behavior are associated with physical fitness and blood lipid levels in primary school children in consideration of gender disparity remains unclear.

**Methods:**

Data was obtained from a health and nutrition survey on primary school children from nine areas in China. The association between physical activities/sedentary behaviors (school and leisure-time physical activity levels, screen time, and other sedentary behaviors) and anthropometric measurements/prevalence of dyslipidemia were examined by multilevel analysis (the individual level, class level, grade level, and investigation area level) adjusted for age, energy intake and family income.

**Results:**

A total of 770 participants (average age = 9.4 ± 1.7 years) were included. Prevalence of dyslipidemia was 10.9%. Prevalence of dyslipidemia was associated with screen time in boys [OR = 3.04, 95% CI (1.24–7.45)] and inversely associated with leisure-time physical activity in boys [OR = 2.22, 95% CI (1.08–4.56)] and school-time activity in girls [OR = 5.34, 95% CI (1.18–24.16)].

**Conclusions:**

Physical activity—but not sedentary behavior—was significantly associated with dyslipidemia in both genders. Increasing leisure-time physical activity for boys and school-time physical activity for girls may be critical.

## Background

It is well known that regular physical activity has numerous health benefits [1–3]. Both biological and psycho-social health benefits of physical activity have been observed in school-age children [4]. In view of school-age children’s daily life, school time and after-school periods are two crucial times where children obtain the majority of their daily physical activity. Thus, both home and school are considered to be critical settings for promoting physical activities [5]. However, how the physical activity practiced in the two periods contributes to health benefits has not been clarified.

On the other hand, with the development of technology, increasing time engaged in sedentary behavior has been noted in children. It is reported that even children who meet physical activity guidelines spend a large proportion of their day engaged in sedentary behavior [6]; high levels of sedentary behavior may coexist with a high total level of physical activity. Accumulating evidence suggests that sedentary behavior, independent of physical activity levels, may be associated with an increased risk of metabolic syndrome, cardiovascular disease, and a variety of other health problems [6–8]. However, a recent review reported a challenging finding: that the relationship between childhood sedentary behavior and biomedical health is unconvincing [9]. It is possible that the influence of sedentary behavior on the development of metabolic health could differ across gender and type of sedentary behavior [10–12]. A recent review suggested an association between children’s screen-time behaviors and metabolic health, which suggested the necessity of evaluating screen-time separately [7].

Moreover, a recent study by Majid et al. carried out in Malaysian adolescents aged from 13 to 15 years old indicated a gender gap between physical activity levels and body composition and, accordingly, blood lipids [13]; Therefore, it is important to take into account of different types of physical activities and the gender gap when assessing its association with the prevalence of dyslipidemia. In the current study, we investigated physical activities and sedentary behaviors in different domains in primary school children, and assessed their association with physical fitness and the prevalence of dyslipidemia, stratified by gender.

## Methods

### Participants

Primary school children in China participated in a cross-sectional study conducted between November 2011 and April 2012. They were selected by a multistage cluster sampling strategy. This study was conducted to assess nutrient intake, physical activity, other lifestyle behaviors, and their association with physical development in children. In the first stage, we selected seven urban areas (Beijing, Guangzhou, Chengdu, Shenyang, Suzhou, Lanzhou, and Zhengzhou) and two rural areas (a lowland area and a mountainous area, both in Hebei province) to reflect different geographies and economic levels. The seven urban areas were from seven different administrative regions, including North China, Southern China, Southwest China, Northeast China, East China, Northwest China, and central China. The two rural areas were from central China. One primary school in each area was selected. From each participant school, one second-grade class and one fifth-grade was randomly chosen (in the case where the number of students in one class was less than 40, two or more classes were randomly selected to achieve a total number of 40 students). All eligible children in the selected classes were investigated. The inclusion and exclusion criteria were as follows: children in the selected classes without reported birth defects (including congenital heart disease, hydrocephalus, and deformity at birth), infantile paralysis and thalassemia, or acute health problems (including the common cold and diarrhea) at the time of the survey were included.

This study was approved by the Ethical Committee of the Health Science Center at Peking University (NO.IRB00001052-11042). Written informed consent forms were collected from the legal guardians of all participants.

### Questionnaire survey

Questionnaire surveys were given to the legal guardians and school teachers of participants because of the young age of the participants; this is a common approach according to a recent review [6]. We collected relevant information including gender, age (accurate to date), area in which participants lived, physical activity levels and sedentary behaviors, energy intake and family income per capita. Leisure-time and school-time activity levels were investigated separately. The legal guardians were asked about their children’s frequency of engagement in and average time spent on leisure-time activity items per week. The items included running, swimming, riding bicycles, playing football, basketball, table tennis, badminton, dancing, climbing mountains, skating, rope skipping, playing hopscotch, rubber band skipping, beanbag games, other group games (chasing or romping), and other physical activities. The class teachers were asked about the frequency and duration of physical education classes and national united broadcasted callisthenics at break-time per week. The duration of leisure-time sedentary behaviors (① watching TV;② watching video, VCD, or DVD; ③ using a computer; ④ using a cell phone; ⑤ reading, writing, or drawing; ⑥ sedentary games including playing chess or playing with toys or dolls while sitting; and other sedentary behaviors) accurate to 0.5 h per day was provided by the legal guardians. The food intake during the previous 24 h of the survey was investigated by a 24-h dietary recall and total energy intake was calculated from the food items reported.

In consideration of the numbers of participants in each category and with reference to the reported average time of sedentary behavior [14], we classified total leisure-time sedentary activity level as high (any of the sedentary behaviors ≥1.0 h/day) or low (all of the sedentary behaviors <1.0 h/day). Subsequently, we classified screen time (① watching TV; ② watching video, VCD, or DVD; ③ using a computer; ④ using a cell phone) and other leisure-time sedentary activities (① reading, writing, or drawing; ② sedentary games) using the same criteria. We then multiplied frequency by duration and sum across physical activities to obtain total physical activity time per week. We stratified the total level of physical activity into leisure-time physical activity and school-time physical activity by the lowest quartile (i.e., the lowest 25% vs. the highest 75%).

### Anthropometric measurements

Anthropometric measurements were carried out by trained investigators using a standardized protocol. Participants wore minimal clothing; i.e., they removed all heavy clothes and shoes. Height was measured to the nearest 0.1 cm, using a height measuring tape suspended from the wall, and weight was measured to the nearest 0.1 kg by a calibrated electronic weighing scale. Waist circumference (WC) and hip circumference (HC) were measured using a flexible and inelastic tape. WC was measured at 2 cm above the umbilicus according to the standard by Yan el al. [15]. HC was measured at maximal protrusion of the buttocks. All of these anthropometric variables were accurate to 0.1 cm. Body mass index (BMI) was calculated as weight/height^2^ (kg/m^2^). BMI z-score was calculated using the criteria of the World Health Organization. Waist-hip ratio (WHR) and waist-height ratio (WHtR) were calculated as WC/HC and WC/height, respectively.

### Blood lipid profiles

Venous blood samples were collected following an overnight fast. Total cholesterol (TC), triglycerides (TG), and high density lipoprotein-cholesterol (HDL-C) were assayed by enzymatic methods using an autoanalyzer (Modular P-800; Roche, Switzerland). The concentration of low density lipoprotein-cholesterol (LDL-C) was calculated using the Friedewald equation (LDL ‐ C = TC ‐ (HDL ‐ C + TG/5)).

Dyslipidemia was defined according to the National Cholesterol Education Program [16] as well as the Chinese journal publication “Experts Consensus for Prevention and Treatment of Dyslipidemia in Children and Adolescents” [17]. The cut-off values of dyslipidemia were as follows: TC ≥ 200 mg/dL (5.172 mmol/L), LDL-C ≥ 130 mg/dL (3.3618 mmol/L), TG ≥ 150 mg/dL (1.6935 mmol/L), or HDL-C ≤ 35 mg/dL (0.9051 mmol/L).

### Statistical methods

All statistical analysis was conducted using SAS version 9.3 (SAS Institute Inc., Cary, NC, USA). All participants with anthropometric data, blood lipid data, and physical activity data were included in the analysis. Basic characteristics between genders were compared using t-tests for continuous variables and the Rao-Scott *χ* ^2^ test for categorical variables (clustering in class level and strata in grade level and investigation area level were considered). We first examined whether sedentary behavior/physical activity were associated with BMI z-score, WHR, or WHtR using multilevel analysis by the MIXED procedure (a linear multilevel analysis with continuous outcome variables). Subsequently, we examined the association between sedentary behavior/physical activity and the prevalence of dyslipidemia using a similar four-level model using the GLIMMIX procedure (a logistic multilevel analysis with dichotomous outcome variables). A four-level structure was constructed for all models; i.e., participants clustered within class, classes clustered within grade, and grades clustered within investigation area (equivalent to schools since only one school was selected in each investigation area). A random intercept was considered for each level. In Model 1, we adjusted for age, total energy intake per day, and family income per capita. In Model 2, we further included both physical activity and sedentary behavior.

## Results

From a total of 931 participants, 770 participants with complete information on anthropometric measurements, blood lipid levels, and physical activity data were included in the analysis. Of these participants, 398 were boys and 372 were girls; 76% were from urban areas and 24% were from rural areas. The average age of the participant children was 9.4 ± 1.7 years old. Basic characteristics by gender are indicated in Table 1. Boys showed higher BMI z-score, WHR, and WHtR than girls. Boys were found to be more likely to spend less than 60 mins/week in leisure-time physical activity; girls were more likely to spend less than 100 mins/week in school-time physical activity.

**Table 1:** Basic characteristics of participants by gender

	Boys	Girls
Number of participants, n	398	372
Average age, year (mean ± SD)	9.3 ± 1.8	9.4 ± 1.7
Living areas, n		
Urban	**311**	**272**
Rural	**87**	**100**
Anthropometric measurements (mean ± SD)		
Height, cm	136.1 ± 11.2	136.5 ± 11.4
Weight, kg	33.1 ± 11.2	32.1 ± 10.0
BMI z-score	**0.24 ± 1.53**	**−0.05 ± 1.33**
WHR	**0.84 ± 0.06**	**0.821 ± 0.06**
WHtR	**0.45 ± 0.05**	**0.44 ± 0.04**
SB, n		
Screen time^a^		
Any item ≥1.0 h/day	**172**	**139**
All items <1.0 h/day	**226**	**233**
Other leisure-time SB^b^		
Either item ≥1.0 h/day	121	121
both items <1.0 h/day	277	251
PA, n		
Leisure-time PA^c^		
< 60 mins/week (lowest 25%)	**86**	**59**
≥ 60 mins/week (highest 75%)	**254**	**266**
School-time PA^d^		
< 100 mins/week (lowest 25%)	**51**	**71**
≥ 100 mins/week (highest 75%)	**276**	**235**
Blood lipid profiles, mmol/L (mean ± SD)		
TC	3.84 ± 0.64	3.86 ± 0.67
TG	0.97 ± 0.55	1.02 ± 0.57
HDL-C	1.47 ± 0.34	1.44 ± 0.31
LDL-C	1.87 ± 0.51	1.89 ± 0.55
Prevalence of dyslipidemia, n	47	37

Significant difference between groups were marked in bold. Differences between genders were calculated by t-tests for continuous variables and Rao-Scott *χ* ^2^ test for categorical variables (clustering in class level and strata in grade level and investigation area level were considered)

*SB* sedentary behavior, *PA* physical activity

^a^screen time includes ① Watching TV; ② Watching video, VCD, or DVD; ③ Using a computer; ④ Using a cell phone

^b^Other leisure time SB includes ① Reading, writing, drawing; ② Playing chess, playing with toys or dolls while sitting; and other sedentary behaviors

^c^Leisure-time PA includes running, swimming, riding bicycles, playing football, basketball, table tennis, badminton, dancing, climbing mountains, skating, rope skipping, playing hopscotch, rubber band skipping, beanbag games, other group games (chasing or romping), and other physical activities

^d^School-time PA includes physical education classes and calisthenics during the break

The association between sedentary behavior/physical activity and anthropometric measurements is presented in Table 2. For both genders, no significant association between sedentary behavior/physical activity and BMI z-score/WHR/WHtR was observed. Different types of sedentary behavior/physical activity in primary school children and their association with prevalence of dyslipidemia are shown in Table 3. High levels of sedentary behavior—specifically high levels of screen time—were associated with increased risk of dyslipidemia in boys [OR = 3.04, 95 % CI (1.24–7.45)]. Furthermore, low levels of leisure-time [OR = 2.22, 95% CI (1.08–4.56)] and total physical activity [OR = 2.78, 95 % CI (1.27–6.08)] were significantly associated with increased prevalence of dyslipidemia in boys. In contrast, less school-time physical activity was significantly associated to an increased risk of dyslipidemia in girls [OR = 5.34, 95% CI (1.18–24.16)]. After including both physical activity and sedentary behavior in the model, the effect of total leisure time sedentary behavior was attenuated. The effect of screen time in boys and physical activity in both genders did not vary largely.

**Table 2:** Association between sedentary behavior^a^/physical activity^b^ and anthropometric measurements

	BMI z-score Mean ± SD	WHR Mean ± SD	WHtR Mean ± SD
Boys			
SB			
Any item ≥1.0 h/day vs. All items <1.0 h/day			
Regression coefficient (95% CI, model 1^c^)	0.222 (-0.072–0.517)	0.013 (-0.001–0.027)	0.009 (-0.003–0.020)
Regression coefficient (95% CI, model 2^d^)	0.234 (-0.122–0.589)	0.012 (-0.003–0.028)	0.010 (-0.003–0.022)
Total PA			
< 200 mins/week (lowest 25%) vs. ≥200 mins/week (highest 75%)			
Regression coefficient (95% CI, model 1^c^)	0.060 (-0.291–0.412)	0.010 (-0.005–0.026)	0.011 (-0.002–0.023)
Regression coefficient (95% CI, model 2^d^)	0.051 (-0.301–0.402)	0.010 (-0.003–0.028)	0.010 (-0.002–0.023)
Girls			
SB			
Any item ≥1.0 h/day vs. All items <1.0 h/day			
Regression coefficient (95% CI, model 1^c^)	0.203 (-0.075–0.481)	0.005 (-0.008–0.019)	0.004 (-0.006–0.014)
Regression coefficient (95% CI, model 2^d^)	0.287 (-0.060–0.634)	0.003 (-0.013–0.018)	0.005 (-0.006–0.016)
Total PA			
< 200 mins/week (lowest 25%) vs. ≥200 mins/week (highest 75%)			
Regression coefficient (95% CI, model 1^c^)	−0.075 (-0.457–0.307)	−0.001 (-0.017–0.016)	−0.005 (-0.018–0.007)
Regression coefficient (95% CI, model 2^d^)	−0.063 (-0.444–0.319)	0.001 (-0.016–0.017)	−0.005 (-0.017–0.007)

*SB* sedentary behavior, *PA* physical activity

^a^sedentary behavior includes screen time (① Watching TV; ②Watching video, VCD or DVD; ③Using a computer; ④Using a cell phone) and other leisure time SB includes ① Reading, writing, drawing; ② Playing chess, playing with toys or dolls while sitting; and other sedentary behaviors

^b^Total PA includes leisure-time PA (running, swimming, riding bicycles, playing football, basketball, table tennis, badminton, dancing, climbing mountains, skating, rope skipping, playing hopscotch, rubber band skipping, beanbag games, other group games (chasing or romping), and other physical activities) and school-time PA (physical education classes and calisthenics during the break)

^c^Model 1 was constructed by multilevel analysis (the individual level, class level, grade level, and investigation area level), adjusted for age, total energy intake per day, and family income

^d^Model 2 was the same as Model 1, but additionally adjusted for PA when association for SB was examined, and vice versa

**Table 3:** Association between sedentary behavior^a^/physical activity^b^ and prevalence of dyslipidemia in primary school children

	*n*	Dyslipidemia in all children *n*(%)	Adjusted OR of dyslipidemia in all children	Adjusted OR of dyslipidemia in boys	Adjusted OR of dyslipidemia in girls
SB					
Screen time					
Any item ≥1.0 h/day	315	43			
model 1^c^			**1.65 (1.01–2.71)**	**3.01 (1.51–5.98)**	0.94 (0.41–2.18)
model 2^d^			**2.11 (1.11–3.99)**	**3.04 (1.24–7.45)**	1.83 (0.64–5.29)
All items <1.0 h/day (ref.)	465	42	ref.	ref.	ref.
Other leisure time SB					
Either item ≥1.0 h/day	244	26			
model 1^c^			0.90 (0.53–1.54)	1.39 (0.72–2.70)	0.51 (0.20–1.35)
model 2^d^			1.01 (0.53–1.92)	1.19 (0.52–2.71)	0.89 (0.29–2.75)
both items <1.0 h/day	536	60	ref.	ref.	ref.
Total leisure time SB					
High (Any item ≥1.0 h/day)	439	51			
model 1^c^			1.14 (0.68–1.92)	**2.57 (1.20–5.50)**	0.50 (0.22–1.15)
model 2^d^			1.61 (0.80–3.26)	2.68 (0.95–7.61)	1.13 (0.36–3.51)
Low (All items <1.0 h/day)	341	34	ref.	ref.	ref.
PA					
Leisure-time PA					
< 60 mins/week (lowest 25%)	149	19			
model 1^c^			1.64 (0.90–2.97)	**2.27 (1.11–4.64)**	0.72 (0.20–2.66)
model 2^d^			1.62 (0.89–2.96)	**2.22 (1.08–4.56)**	0.72 (0.20–2.65)
≥ 60 mins/week (highest 75%)	522	51	ref.	ref.	ref.
School-time PA					
< 100 mins/week (lowest 25%)	122	21			
model 1^c^			**2.94 (1.26–6.88)**	2.34 (0.80–6.87)	**6.26 (1.21–32.42)**
model 2^d^			**3.30 (1.39–7.84)**	2.80 (0.93–8.44)	**5.34 (1.18–24.16)**
≥ 100 mins/week (highest 75%)	519	46	ref.	ref.	ref.
Total PA (model 1^c^)					
< 200 mins/week (lowest 25%)	133	19			
model 1^c^			1.86 (0.99–3.47)	**2.79 (1.29–6.05)**	0.80 (0.21–2.98)
model 2^d^			1.85 (0.99–3.48)	**2.78 (1.27–6.08)**	0.80 (0.21–3.01)
≥ 200 mins/week (highest 75%)	426	36	ref.	ref.	ref.

Significant difference between groups were marked in bold

*SB* sedentary behavior, *PA* physical activity

^a^sedentary behavior includes screen time (① Watching TV; ② Watching video, VCD, or DVD; ③ Using a computer; ④ Using a cell phone) and other leisure time SB includes ① Reading, writing, drawing; ② Playing chess, playing with toys or dolls while sitting; and other sedentary behaviors

^b^Total PA includes leisure-time PA (running, swimming, riding bicycles, playing football, basketball, table tennis, badminton, dancing, climbing mountains, skating, rope skipping, playing hopscotch, rubber band skipping, beanbag games, other group games (chasing or romping), and other physical activities) and school-time PA (physical education classes and calisthenics during the break)

^c^Model 1 was constructed by multilevel analysis (the individual level, class level, grade level, and investigation area level), adjusted for age, total energy intake per day, and family income

^d^Model 2 was the same as Model 1, but additionally adjusted for PA when association for SB was examined, and vice versa

## Discussion

This study firstly examined physical activity in different domains and sedentary behavior in children at nine Chinese primary schools. Subsequently, we assessed the association between sedentary behavior/physical activity and anthropometric indicators/prevalence of dyslipidemia. As a result, associations between physical activity/sedentary behavior and dyslipidemia were not consistently found for all domains. Screen time—but not other leisure-time sedentary behavior—was identified to be associated with higher prevalence of dyslipidemia in boys. Recent reviews have demonstrated that screen time is a crucial factor influencing cardiovascular health indicators [6, 8] and an international study indicates that boys engage in more screen time than girls [18].

This current study found that leisure-time and school-time physical activity were associated with an increased risk of dyslipidemia in boys and girls, respectively. A series of previous studies show consistent results that physical activity is associated with cardiovascular risk factors in children and youth [19–21]. This study demonstrates similar results, although the results are not consistent in all domains. We found that reduced leisure-time physical activity is associated with increased risk of dyslipidemia in boys while low school-time activity is associated with high prevalence of dyslipidemia in girls. A study by Stanley et al. indicates that the content and intensity of physical activity differs between genders [22]. The intensity and duration of physical activity may influence the health outcomes every time. However, this information was not investigated in this study. Further studies are needed to clarify the possible explanation of the gender disparity.

This study suggests an attenuated effect of total sedentary behavior in boys after adjustment for physical activity, but not vice versa. This was consistent with a previous study by Garcia-Hermoso et al. [23]. In that study, the authors indicated that the effects of sedentary behavior on cardiovascular risk factors are mediated by physical activity. Our results confirmed this hypothesis to a certain degree. However, we could not rule out the possibility that the insignificant association was because of our limited sample size.

There are some limitations which need to be addressed in this study. First, information on physical activity is limited to duration and frequency in this study. Although we may speculate about intensity according to the content of the physical activity, there might be great individual variation. Second, sedentary behavior was investigated as accurate to 0.5 h. Therefore, accurate sedentary time per day was not available for this study. We had to use categorical sedentary time for analysis. Third, reporting bias may exist since the questionnaire survey was applied to the legal guardians and school teachers due to the young age of the participants. Last, the current study is cross-sectional and sample size of this study is limited, so we have to be cautious when drawing conclusions.

## Conclusions

This study indicates that in both genders dyslipidemia is significantly associated with physical activity, but not sedentary behavior. Increasing leisure-time physical activity for boys and school-time physical activity for girls may be critical. However, further large-scale studies are needed to confirm these findings before intervention programs could be performed.

## Abbreviations

BMI: Body mass index
HC: Hip circumference
HDL-C: High density lipoprotein-cholesterol
LDL-C: Low density lipoprotein-cholesterol
PA: Physical activity
SB: Sedentary behavior
TC: Total cholesterol
TG: Triglycerides
WC: Waist circumference
WHR: Waist-hip ration
WHtR: Waist-height ratio
